# How the physical appearance of companions affects females with high or low social physique anxiety: a virtual reality exercise study

**DOI:** 10.1007/s10055-022-00676-w

**Published:** 2022-07-25

**Authors:** Rianca Kroon, David L. Neumann, Timothy M. Piatkowski, Robyn L. Moffitt

**Affiliations:** 1 School of Applied Psychology, Griffith University, Brisbane, QLD 4222 Australia; 2 College of Health and Human Sciences, Charles Darwin University, Casuarina, NT Australia; 3 School of Psychology, Deakin University, Burwood, VIC Australia

**Keywords:** Exercise, Health promotion, Social physique anxiety, Virtual reality

## Abstract

Technologies such as virtual reality (VR), an immersive computer-based environment that induces a feeling of mental and physical presence, are becoming increasingly popular for promoting participation in exercise. The purpose of this study was to explore changes in motivation and other psychological states when the physique of an exercise companion was altered during a VR-based exercise task, and whether trait social physique anxiety (SPA) altered these effects. Using a mixed experimental design, female participants (*N* = 43) categorised as high or low in SPA participated in two counterbalanced 10-min running tasks within a VR environment where the exercise companion was either overweight or in-shape. Across both running tasks, individuals with high SPA reported higher negative affect, pressure and tension, and lower perceived competencies, than those with low SPA. Pressure and tension were also higher when exercising with an in-shape companion than with an overweight companion for all participants. In addition, participants with high SPA reported a stronger preference to exercise with an overweight companion than those with low SPA in a real exercise setting, but not in a VR setting. The findings suggest that the physique of an exercise companion and the SPA of an exerciser have important, but independent, psychosocial effects during exercise. That an in-shape physique of a virtual exercise companion was not a deterrent among those with high SPA has provided preliminary evidence that VR-based exercise may be helpful among females who worry about their appearance or feel self-conscious while exercising.

## Introduction

Researchers have investigated exercise interventions to improve key health markers across many modalities, with limited effectiveness (Ismail et al. 2012; Johns et al. 2014; Ohkawara et al. 2007). A 50% attrition of attendance typically occurs within the first 6 months of a new exercise program and many participants experience partial weight increase over the long term (Wu et al. 2009). The lack of consideration of individual characteristics has been identified as a limitation to many current exercise interventions (Bauman et al. 2012), and it has been acknowledged that individuals have different preferences for exercise settings (Dunlop and Schmader 2014; Pearson et al. 2013). Social physique anxiety (SPA), a feeling of distress associated with the perceived evaluation of one's physical self, is one such characteristic. SPA may discourage individuals, particularly females, from exercising in the presence of others. The present study examined the effect of SPA on self-reported exercise preferences in a real-world and virtual reality (VR) setting. In addition, we explored the effects of SPA and the physique of an exercise companion on the motivation and affective state of female exercisers when exercising in a VR environment.

Social presence and support in the exercise environment have positive implications for exercise adherence (Fraser and Spink 2002; McAuley et al. 2000). Social support is the perception of assistance in performing the exercise behaviour, which can be implemented through buddy systems, spousal participation, encouragement, and positive feedback from leaders and peers (Rhodes 2006). Lower levels of exercise compliance have been demonstrated in programmes that are individually driven in comparison to those with group interaction and participation (Fraser and Spink 2002). Indeed, participants have reported walking with a companion to be the most important preference within the social context (Cohen-Mansfield et al. 2004). Exercising with a partner improves adherence and psychological states by providing motivational, social, and instructional support (Rackow et al. 2015). Plante et al. (2011) investigated the psychological benefits of social contact during exercise and found that individuals who felt calm with their exercise companion reported higher enjoyment of the exercise than those who exercised alone. Moreover, the inclusion of social companions specifically within virtual exercise settings has produced changes in both psychological and physiological outcomes (Murray et al. 2016; Neumann et al. 2017).

Festinger’s (1954) Social Comparison Theory (SCT) provides some insight into the possible mechanisms underlying these social effects during exercise. The theory suggests that individuals gain information about themselves through personal comparisons with those they perceive to be better on an important dimension. Such upward comparison can promote exaggerated negative self-perceptions that are disproportionate to the reality of the situation, a phenomenon known as the negative contrast effect (Cash et al. 1983; Thornton and Maurice 1999). This effect has been shown to contribute to body anxiety and dissatisfaction, and changes in exercise and diet in females who compare themselves unfavourably against body ideals (Mischner et al. 2013).

A salient personality characteristic that can increase the likelihood of a negative contrast is SPA. SPA is a subtype of social anxiety, which represents the concerns, thoughts, and feelings that others are evaluating an individual’s physique in a negative way (Crawford and Eklund 1994). SPA is negatively related to exercise adherence (Dunlop and Schmader 2014; Pearson et al. 2013) and can function as a strong barrier to exercise in exercise settings that induce heightened concerns of having one’s physique evaluated by others (Lindwall and Lindgren 2005). Although research has explored variables related to SPA and their connection with exercise behaviours (e.g. Pearson et al. 2013), little research exists on the relationship between SPA and preferences for different modalities of exercise.

In one exception, Dunlop and Schmader (2014) investigated the impact of the physique of members in an exercise program as a motivator for individuals presented with various exercise context vignettes. Individuals with high SPA were less motivated by the prospect of exercising with in-shape individuals than those with low SPA. In contrast, low SPA individuals found it more appealing to exercise alongside normal weight individuals. These findings were consistent with aspects of SCT, where those who care little about the evaluation of their physique (low SPA) preferred an in-shape exerciser since it increases motivation and therefore results in an upward comparison. In contrast, downward comparisons were made by individuals with high SPA as they likely felt more susceptible to harsh physique evaluation and threat when contemplating exercise in the presence of an in-shape than an overweight companion (Dunlop and Schmader 2014). Although suggestive of a relationship between SPA and preferences associated with exercise companions, Dunlop and Schmader’s (2014) investigation is limited by the reliance on self-reports and use of exercise vignettes.

One way to explore the effect of the physique of an exercise companion during an actual rather than imagined exercise task, and in a controlled social context, is with VR. VR is a computer-based environment that induces a feeling of mental and physical presence and can create social presence in an exercise programme by the introduction of avatars of exercise companions (e.g. Lee et al. 2013; Nunes et al. 2014). Several studies have demonstrated the efficacious use of VR-based exercise programmes to improve coping during COVID-19 in older adults (Gao et al. 2020), to increase motivation and enjoyment of exercise for college students (Zeng et al. 2017), and for improving physical fitness in individuals with haemodialysis (Cho and Sohng 2014) and intellectual or developmental disabilities (Lotan et al. 2009). The implementation of VR-based exercise programmes within gyms or home settings is becoming more feasible with the increasing availability of VR apps and commercially produced software (Neumann et al. 2017). Such VR programmes utilise a range of equipment such as lightweight head-mounted displays, allowing for a fully immersive virtual environment. This equipment is integrated with sensors to synchronise the movement of exercisers and exercise equipment with virtual movements, such as a bicycle moving through a virtual environment (Ng et al. 2019). Preliminary evidence suggests that the presence of avatars representing virtual others in conjunctive (Irwin et al. 2012) or competitive (Murray et al. 2016; Neumann et al. 2017) situations can influence the performance and psychological states of exercisers. Thus, social comparisons appear to be a salient factor when exercising in a VR environment. Although valuable findings have emerged from studies that have investigated SPA and VR-based exercise based independently, no research to date has investigated the psychological impacts of VR-based exercise amongst individuals with varying levels of SPA within the same study.

In an extension of previous work in this area, the present study examined the motivational and affective states of exercisers during real rather than imagined exercise, in a controlled VR-based exercise setting. Based on findings that SPA is more prevalent in females than in males (Kowalski et al. 2006; Kruisselbrink et al. 2004), a female participant sample with varying levels of SPA was recruited. Participants completed two running exercise tasks in a VR environment in the presence of an avatar that represented one of two exercise companions. In real life, one companion was in-shape, and the other was overweight. It was hypothesised that women high in SPA would report lower motivation, lower positive affect, and higher negative affect than women low in SPA across both running tasks. We also expected lower motivation, lower positive affect, and higher negative affect when exercising with an in-shape companion than an overweight companion. However, the negative effect of the presence of an in-shape exercise companion was expected to be more pronounced among the high SPA group. We further hypothesised that high SPA individuals would report a stronger preference to exercise with an overweight than an in-shape exercise companion. In an exploratory investigation, we compared high SPA and low SPA exercisers preferences for an in-shape or overweight exercise companion across real-world and VR exercise settings. The findings of the present study will potentially inform the suitability of avatar-based VR exercise programmes for females who worry about their appearance, particularly within exercise settings, as a means to increase physical activity and improve psychological states.

## Method

### Participants

The study used a mixed SPA (high, low) × exercise companion (in-shape, overweight) experimental design; participants were categorised into high or low SPA groups and completed two counterbalanced running tasks with an in-shape or overweight exercise companion. In the study reported by Dunlop and Schmader (2014), the effect size for the interaction between  SPA and member composition of exercise companion was *η*_*p*_^2^ = 0.10. Using this effect size in an a priori power analysis using G*Power (Faul et al. 2007) with Power = 0.95 and *α* = 0.05 yielded a minimum sample size of 32 to detect an interaction effect in the present 2 × 2 factorial design. A total of 45 female undergraduate students and members of the community were recruited, although two participants failed to provide data for the second time point. The final sample comprised 43 females, 40 from the undergraduate sample and 3 from the community sample (*M *Age = 22.19, *SD* = 5.67). All participants were given the opportunity to enter a draw to win a gift voucher as reimbursement for their time. The study received approval from Griffith University’s Human Research Ethics committee prior to data collection.

### Apparatus

**Fig. 1:**
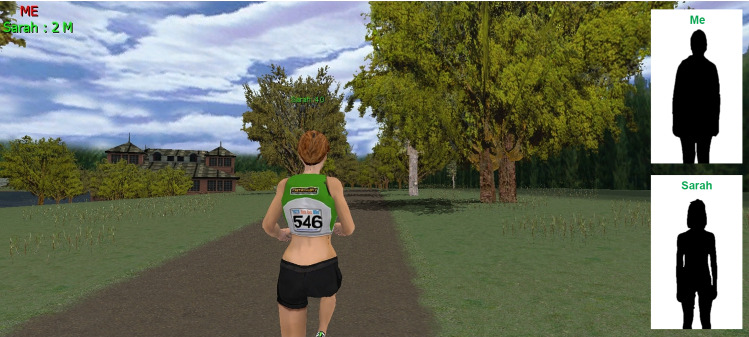
Example screenshot of the virtual reality environment depicting the virtual track, landscape, and buildings and the avatar of the companion exerciser. Colour photographs of the research participant (“Me”) and the confederate companion exerciser (“Sarah”) were overlaid on the virtual environment as indicated (silhouettes are shown to preserve anonymity)

The experiment was conducted in a climate-controlled room with a light intensity of 10.1 lx. The VR environment, generated by the Netathlon 2 XF software, was projected through a BenQ MW870UST projector as a 2.5 m × 1.35 m image on a white wall. Participants ran on a Marquee Fitness MT80 treadmill, which was placed 1.2 m from the wall. The treadmill was interfaced with the VR software via an ANT + receiver, allowing for the participants speed on the treadmill to match the projected image. Participants had a third-person view, allowing them to view the exercise companion’s avatar throughout the course. The exercise route used was the Head of the Charles which depicted scenery of trees, bridges, buildings, and a gentle river. An example screenshot of the virtual environment and avatar of the companion exerciser is shown in Fig. 1. A colour photograph of the participant was taken prior to the task and overlaid on the virtual environment, along with a photograph of the confederate companion exerciser. All measures of distance, speed, incline, and heart rate (HR) from the treadmill and VR were visually covered throughout the experiment to prevent real-time feedback on performance from confounding the results. Whilst running, HR was measured using an electrocardiograph by placing electrodes over the chest region. The signal was sent to a 4/20 PowerLab data acquisition system via an Acumen receiver.

### Measures

Demographic information: Demographic information was collected as well as weight (in kilograms) and height (in centimetres) to measure participant BMI (*M* = 23.20, *SD* = 4.08). Participants were screened for medical risk, and no participants were excluded on this basis (Sports Medicine Australia 2005). Levels of physical activity were measured via the International Physical Activity Questionnaire—Long Form (IPAQ-LF; Craig et al. 2003) with participants scoring in moderate (*n* = 17) or high (*n* = 26) categories.

Social Physique Anxiety Scale (SPAS; Hart et al. 1989): This measure contains 12 items measured on a 5-point Likert scale ranging from 1 = *not at all characteristic of me* to 5 = *extremely characteristic of me*. Five of the 12 items were reverse scored and then summed to create a total score (ranging from 12 to 60) where higher scores indicated higher SPA (Hart et al. 1989). The SPAS in the current study demonstrated high reliability (*α* = 0.93).

Positive and negative affect scale (PANAS; Watson et al. 1988): Participants rated 20 emotive descriptive words on a 5-point Likert scale based on how they felt in the present moment, ranging from 1 = *very slightly or not at all* to 5 = *extremely* (Watson et al. 1988). The scale comprised two dimensions of positive and negative affect, and by summing responses, a higher score was indicative of stronger affect (Watson et al. 1988). The current data demonstrated satisfactory reliability for the negative (overweight condition *α* = 0.77 and in-shape condition *α* = 0.82) and positive subscales (overweight condition *α* = 0.90 and in-shape condition *α* = 0.91).

Physical activity enjoyment scale (PACES; Kendzierski and DeCarlo 1991): Participants were asked to rate how they felt in that moment regarding the exercise they had just completed utilising a 7-point semantic differential scale (For example, questions include: 1 = *I find it pleasurable* to 7 = *I find it unpleasurable*, or 1 = *I enjoy it* to 7 = *I hate it*). Eleven of the 18 items were reverse scored and then all items summed, where a higher score indicated greater enjoyment of the physical activity. The present data showed high levels of internal consistency (overweight condition *α* = 0.90 and in-shape condition *α* = 0.95).

Intrinsic motivation inventory (IMI; Ryan 1982): The IMI is a 22-item measure with four scales comprising five items each: perceived competence, effort, pressure/tension, and value. The IMI scale ranges from 1 to 5, where 1 = *not true at all* and 5 = *very true*. For each subscale, the respective five items were scored (reverse scored for pressure/tension) and summed, with a higher score indicating greater perceived competence, effort, value and decreased pressure and tension. The reliability of the subscales for the present data were adequate: perceived competence (overweight condition *α* = 0.81 and in-shape condition *α* = 0.86), effort (overweight condition *α* = 0.85 and in-shape condition *α* = 0.90), pressure and tension (overweight condition *α* = 0.76 and in-shape condition *α* = 0.77) and value (overweight condition *α* = 0.91 and in-shape condition *α* = 0.91).

Preference of exercise setting and physique of exercise companions: Four items were specifically developed for the present investigation to measure participant preferences to exercise with an overweight companion in a VR and a real exercise setting (e.g. “*In a virtual reality setting* [*in a real exercise setting*]*, I would prefer to not exercise alongside a companion who is in-shape rather than overweight* [*who is overweight rather than in-shape*]”). The items in the preference measure drew on previous research methodologies which used VR. For example, Botella Arbona et al. (2014) measured preference for VR exposure and in-vivo exposure therapy and reported a higher preference for VR exposure. Adapting the methods of Botella Arbona et al. (2014), participant preferences in this study were measured using items that presented a dichotomous choice between an overweight and in-shape companion. The level of agreement with the items was rated on a 5-point Likert scale ranging from 1 = *not at all true* to 5 = *very true*. Items asking about preference for an in-shape companion were reverse scored so that higher scores represented a stronger preference to exercise with an overweight companion.

### Procedure

Participants completed two sessions on different days. In the first session, participants provided informed consent, completed medical screening, and provided demographic information. Electrodes for HR measurement were fitted and participants completed a 5-min warm-up on the treadmill with no performance information or VR displayed. The warm-up also familiarised participants to the pre-determined exercise intensity. This was calculated based on HR using the following formulae: 0.7*(208–0.7*age) for 70% HRmax and 0.75*(208–0.7*age) for 75% HRmax, which is classified as the low end  of high-intensity exercise (Norton et al. 2010). The participant next completed the IPAQ-LF. It was next explained that another participant (a confederate) was completing the same study at another campus. The researcher made a phone call in front of the participant to ensure both were ready to commence the exercise. The participant was introduced to one of two confederates, known as exercise companions, Sarah, with a BMI of 19.8 (in-shape) or Olivia, with a BMI of 26.9 (overweight). The two companions were matched on extraneous variables unrelated to weight that might affect participant perceptions of them (e.g. age, ethnicity).

To reinforce the cover story that participants were exercising with a companion in real-time, a brief video-chat session occurred. This chat session utilised a pre-recorded video of the confederate sharing their name, university program and year of study (if a student), or current occupation and favourite movie, under the guise that the interaction was occurring live (see Irwin et al. 2012). The content for both confederates was similar, so as not to confound the physique manipulation. The participant was then invited to share information about themselves, to which the confederate responded with pre-recorded non-verbals (e.g. slight head nods). A photo was then taken of the participant, which was edited to include their name in red font along with a photo of the confederate and their name in green font. The pictures appeared above the VR environment throughout the exercise activity.

Following the interaction, participants began the running task. The task was 10 min long, commencing from the moment they reached the specific intensity level of 70–75% of HRmax. Throughout the exercise, the avatar of the companion in the projected VR environment appeared to be exercising at the same average speed as the participant, sometimes running ahead and sometimes running behind. In reality, the speed was controlled by the VR software using the UltraRabbit setting. Instructions emphasised that participants own HR controlled the exercise intensity, and that this was the same for both runners to minimise the perception of competition. At the completion of the running task, the electrodes were removed, and participants were given 2 min to recover. The PANAS, PACES, and IMI questionnaires were then administered. On completion of the measures, the participant was thanked and asked to return for a second session of data collection within one week.

The procedure of the second session was identical to the first with the exception of the physique of the exercise companion (in-shape or overweight). The order of the companion across sessions was counterbalanced. At the completion of the running task in the second session, the questionnaires were administered alongside the preference scale and the SPAS. Once completed, participants were debriefed, the purpose of the live exercise companion cover story was explained, and participants were thanked for their participation. Each session was approximately 1 h (60 min) in duration.

### Statistical analysis

The data were analysed using a 2 (SPA: high or low) × 2 (physique: in-shape or overweight avatar) mixed factorial ANOVA for the main dependent variables using SPSS version 23. A cut-off score of 48 on the SPAS was used to categorise participants into high (*n* = 12) and low (*n* = 31) SPA groups. Cut-off scores for the formation of high and low SPA groups have been used in similar work previously (Thompson and Chad 2002). This designation of low or high SPA groups was not absolute referencing to SPA and instead represented a relative reference to the level of SPA experienced by the participants in the current study. Categorising participants into groups based on a cut-off score has been shown to produce comparable results to regression analyses when testing for main effects and interactions, whilst maintaining statistical power (Ferreira et al. 2015; Iacobucci et al. 2015a, 2015b; Zimmerman et al. 2004).

The data were examined for outliers and missing values, with none identified. Levene’s test indicated that the assumption of homogeneity of variance was met. The assumption of normality was partially met, indicated by Shapiro–Wilk’s test, as negative affect scores were non-normal. On exclusion of negative affect scores, the violation of normality was reduced, although insufficiently to be eliminated. However, for ease of interpretation alongside the positive affect scores and lack of adjustment from transformation, negativity scores remained as original.

## Results

### Preliminary analyses

A series of one-way ANOVAs indicated that the two SPA groups did not differ in age or IPAQ-LF MET (see Table 1). A significant difference between SPA groups for BMI was found, where high SPA participants had higher BMI than low SPA participants. However, BMI did not correlate with the outcome measures (psychological states), and thus, to maximise statistical power, BMI was not included as a covariate in the main analyses.

**Table 1:** Descriptive statistics for low social physique anxiety (SPA), and high social physique anxiety (SPA) on IPQ, BMI, and Age

Measure	Low SPA (*n* = 31)		High SPA (*n* = 12)		Total		*F*(1, 41)	*p*
	*M*	*SD*	*M*	*SD*	*M*	*SD*		
Age	21.90	5.19	22.92	6.97	22.19	5.67	0.27	0.605
BMI^a^	22.25	3.45	25.66	3.67	23.20	4.08	6.90	0.012
IPAQ^b^	6,939.55	8,098.17	4,322.17	3,196.91	6,209.11	7,136.59	1.17	0.286

^a^Body Mass Index (BMI)

^b^International Physical Activity Questionnaire (IPAQ): High exercise classification = IPAQ MET > 3000

A manipulation check was performed to ensure that HR of participants across the 10-min task did not differ significantly across physique conditions and SPA groups. A 2 (SPA: high or low) × 2 (physique: in-shape or overweight) × 10 (minutes) mixed ANOVA was conducted. A main effect of minutes was found, *F*(9, 369) = 2.71, *p* = 0.005, *η*_*p*_^2^ = 0.06, which reflected an increase in HR over the task. There was no significant difference in HR between SPA groups or across physique conditions *F*(1, 41) < 0.147, *p* > 0.05, *η*_*p*_^2^ < 0.01, *F*(1, 41) = 0.233, *p* > 0.05 *η*_*p*_^2^ < 0.01.

### Subjective measures

#### Affect and enjoyment

**Fig. 2:**
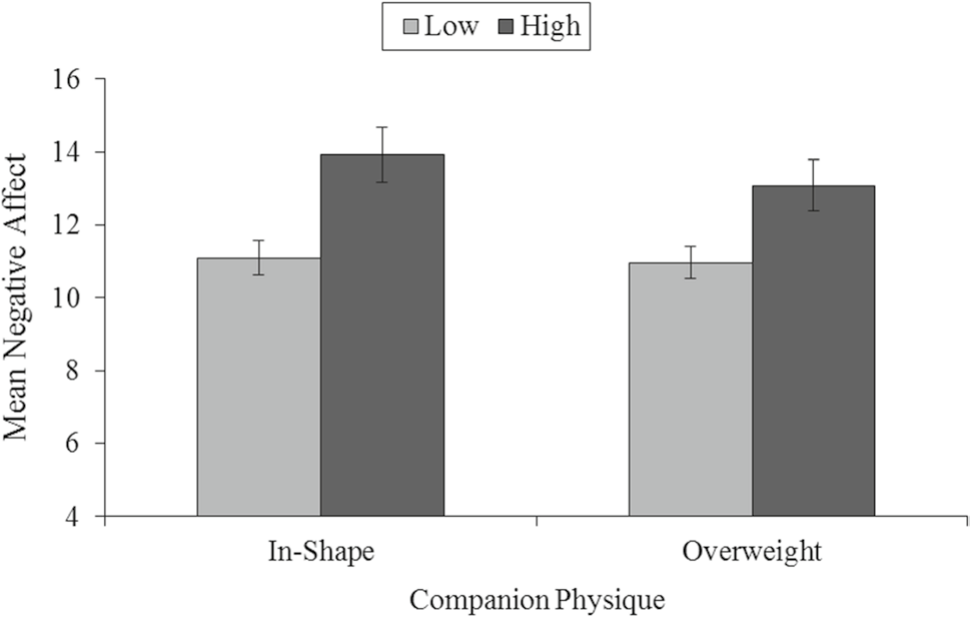
The influence of the physique of an exercise companion for groups with low (*n* = 31) and high (*n* = 12) social physique anxiety (SPA) on mean negative affect. Standard errors are represented in the figure by the error bars attached to each column

No significant main effects or interactions for SPA or physique were found for positive affect, all *Fs*(1, 41) < 0.76, *ps* > 0.05, *η*_*p*_^2^ < 0.02 (see Table 2). A significant main effect of SPA was found for negative affect where high SPA participants reported more negative affect than low SPA participants, *F*(1, 41) = 10.14, *p* = 0.003, *η*_*p*_^2^ = 0.20 (see Fig. 2).There was no significant interaction nor a main effect of physique for negative affect, both *F*(1, 41) < 1.76, *p* > 0.05, *η*_*p*_^2^ < 0.04. No significant interaction or main effects were demonstrated for enjoyment, all *F*(1, 41) < 1.17, *p* > 0.05, *η*_*p*_^2^ < 0.03.

**Table 2:** Descriptive statistics for psychological states for low social physique anxiety (SPA; N = 31) and high social physique anxiety (SPA; *N* = 12), split by physique of exercise companion

Measure	In-shape companion			Overweight companion			Low SPA	High SPA
	Low SPA	High SPA	Total	Low SPA	High SPA	Total	Total	Total
PANAS positive	31.81 (7.20)	31.08 (9.54)	31.60 (7.81)	33.06 (7.00)	31.50 (7.68)	32.63 (7.14)	32.44 (7.10)	31.29 (8.61)
PANAS negative	11.10 (1.35)	13.92 (4.54)	11.88 (2.89)	10.97 (1.17)	13.08 (4.23)	11.56 (2.57)	11.03 (1.26)	13.50 (4.39)
PACES	99.45 (15.69)	94.75 (19.85)	98.14 (16.84)	98.10 (14.77)	98.33 (13.14)	98.16 (14.18)	98.77 (15.23)	96.54 (16.49)
IMI competence	17.71 (2.94)	14.83 (2.98)	16.91 (3.19)	18.32 (3.00)	16.08 (1.56)	17.70 (2.85)	18.02 (2.97)	15.46 (2.27)
IMI effort	19.48 (3.91)	18.83 (4.51)	19.30 (4.04)	19.23 (3.91)	19.33 (3.50)	19.26 (3.76)	19.36 (3.91)	19.08 (4.00)
IMI value	28.55 (5.09)	29.08 (5.45)	28.70 (5.13)	29.13 (4.31)	28.50 (4.06)	28.95 (4.20)	28.84 (4.70)	28.79 (4.75)
IMI pressure tension	9.48 (3.61)	12.25 (4.25)	10.26 (3.95)	8.26 (3.44)	11.00 (3.86)	9.02 (3.73)	8.87 (3.53)	11.63 (8.11)

*PANAS* Positive and Negative Affect Scale, *PACES* Physical Activity Enjoyment Scale, *IMI* Intrinsic Motivation Inventory

#### Intrinsic motivation

**Fig. 3:**
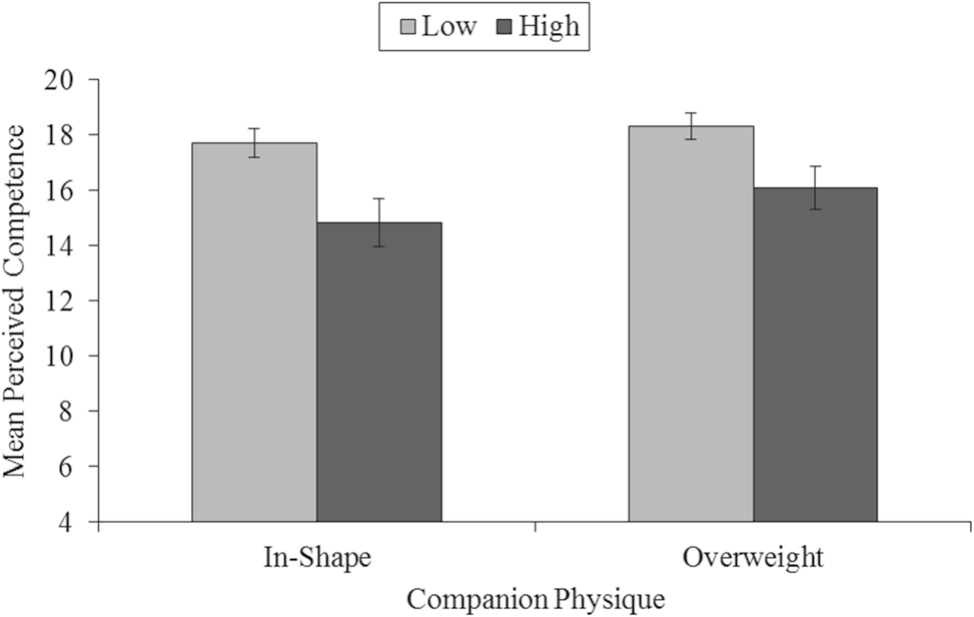
The influence of the physique of an exercise companion for groups with low (*n* = 31) and high (*n* = 12) social physique anxiety (SPA) on mean perceived competence on an exercise task. Standard errors are represented in the figure by the error bars attached to each column

**Fig. 4:**
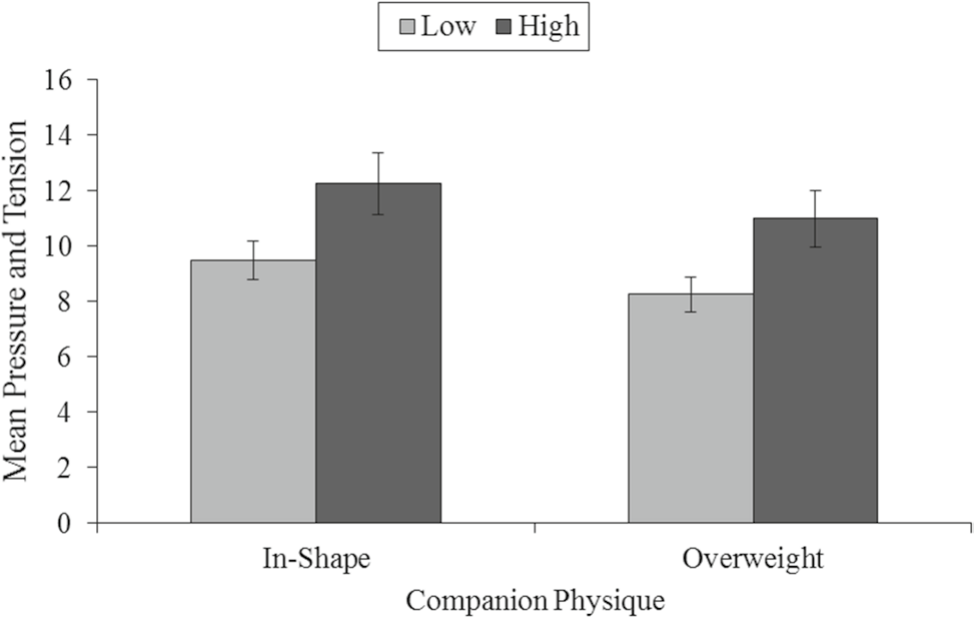
The influence of the physique of an exercise companion for groups with low (*n* = 31) and high (*n* = 12) social physique anxiety (SPA) on mean pressure and tension. Standard errors are represented in the figure by the error bars attached to each column

For perceived competence, there was a main effect for SPA, *F*(1, 41) = 10.70, *p* = 0.002, *η*_*p*_^2^ = 0.21, indicating that participants with low SPA perceived themselves to be more competent at the exercise task than those with high SPA (see Fig. 3). Both the main effect and interaction involving physique were non-significant for perceived competence, *F*(1, 41) = 2.80, *p* = 0.10, *η*_*p*_^2^ = 0.06, and *F*(1, 41) = 0.33, *p* = 0.57, *η*_*p*_^2^ = 0.01, respectively. A moderate to large main effect of both physique, *F*(1, 41) = 6.01, *p* = 0.019, *η*_*p*_^2^ = 0.13, and SPA, *F*(1, 41) = 5.79, *p* = 0.021, *η*_*p*_^2^ = 0.12, was demonstrated for pressure. As shown in Fig. 4, participants reported higher pressure and tension when exercising with an in-shape companion than an overweight companion. Furthermore, individuals with high SPA reported greater pressure and tension than those with low SPA. The interaction between physique and SPA was not significant, *F*(1, 41) = 0.001, *p* = 0.98, *η*_*p*_^2^ = 0.001. No significant interaction or main effects of physique or SPA were observed for effort, all *F*(1, 41) < 1.00, *p* > 0.05, *η*_*p*_^2^ < 0.02, and value, all *F*(1, 41) < 1.11, *p* > 0.05, *η*_*p*_^2^ < 0.03.

#### Physique preference

**Fig. 5:**
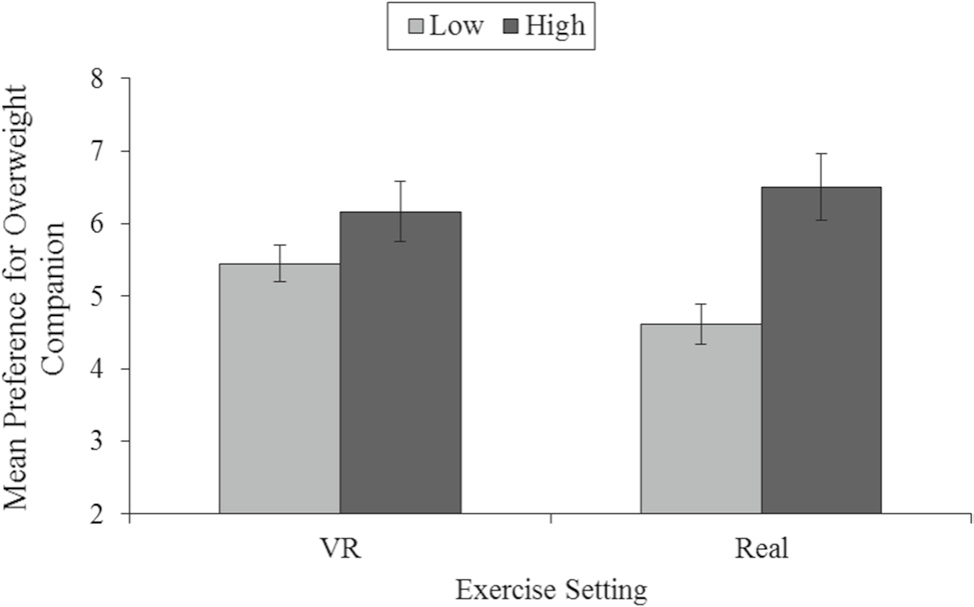
Preference to exercise with an overweight companion for groups with low (*n* = 31) and high (*n* = 12) social physique anxiety (SPA) within a real exercise setting and a virtual reality (VR) exercise setting. Standard errors are represented by the error bars

A one-way ANOVA was conducted for each of the exercise settings to assess the difference between SPA groups on preferences to exercise with an overweight companion. As indicated in Fig. 5, no significant difference between SPA groups was found for physique of companion preferences within a VR setting, *F*(1, 41) = 2.13, *p* = 0.15, *η*_*p*_^2^ = 0.05. A significant difference, with a large effect, was revealed across levels of SPA for preference of companion physique within a real exercise setting, *F*(1, 41) = 30.81, *p* = 0.001, *η*_*p*_^2^ = 0.23. The finding indicates that individuals with high SPA reported a greater preference to exercise with an overweight individual within a real exercise setting than individuals with low SPA.

## Discussion

The purpose of this study was to explore how psychological states, motivation to exercise, and exercise setting preferences varied depending on levels of SPA and the physique of an exercise companion during a VR-based exercise task. The results demonstrate that across both SPA groups, participants reported increased pressure and tension when exercising with an in-shape companion than an overweight companion. Those with high SPA reported greater negative affect, pressure and tension, and lower perceived competence following the exercise task, compared to individuals with low SPA, irrespective of the physique manipulation. A stronger preference to exercise with an overweight companion was also observed for high SPA individuals than low SPA individuals in a real exercise setting. However, preference for an overweight exercise companion did not differ between SPA groups in a VR-based exercise setting.

All participants, regardless of SPA category, reported increased pressure and tension when exercising with an in-shape rather than overweight companion. The present investigation fits with extant literature around psychological states and exercise companions. For example, Plante et al. (2011) demonstrated no differences in psychological states across in-shape and not in-shape exercise companions, although this was in a sample of healthy college students. Ginis et al. (2008) indicated that appearance-focussed exercise environments, with high physique saliency and body focussed encouragement, result in increased negative psychological states amongst those with high SPA. Furthermore, given that the mean BMI of participants for this study (*M* = 23.2) was higher than the in-shape exercise companion’s BMI (19.8), participants were potentially more concerned by the in-shape companion.

Features of VR-based exercise programmes convey a less pronounced display of an individual’s physical appearance, abilities, and movements through the use of avatars. SCT suggests that during a VR task, comparison with others may thus be less pronounced (Festinger 1954). Specifically, for individuals with high SPA, the SCT framework suggests that upward comparisons may contribute to the development of negative psychological states. Self-presentation aspects of exercise settings can predispose individuals with high SPA to negative psychological experiences (i.e. increased state anxiety, negative feeling states and lowered competence), which are more likely to encourage upward comparisons (Focht and Hausenblas 2004; Ginis et al. 2007; Kruisselbrink et al. 2004; Raedeke et al. 2007) through feelings of inferiority, namely the negative contrast effect (Cash et al. 1983; Thornton and Maurice 1999). The current findings build on previous research by demonstrating that increased negative perceptions and affect for both high and low SPA individuals fit within an SCT framework, with the negative contrast effect demonstrating potential for moderating these relationships. The present data suggest that the strength of negative psychological experience, at least in the context of body comparisons, seems to apply to individuals across all levels of SPA and in a VR exercise setting. Given that little previous research has investigated the SCT framework within VR exercise settings, the current study has important implications. The findings show that the SCT framework may help to explain behaviour in VR environments, however, future research is needed to explore this more fully.

Research investigating the motivational mechanisms and self-efficacy processes of exercise reports a negative link between SPA and perceived competence (Thøgersen-Ntoumani and Ntoumanis 2007). Individuals with higher SPA report lowered feelings of exercise competence, which is consistent with the current findings. In high evaluation or socially threatening exercise settings, individuals with high SPA report increased negative psychological states (Focht and Hausenblas 2004; Ginis et al. 2007; Raedeke et al. 2007). The present data have reinforced past findings by demonstrating increased negative psychological experiences for those with SPA engaging in exercise. Despite the proposition for VR-based exercise tasks to better control for the undesirable psychological responses to exercise settings for those with high SPA, this relationship still exists within a VR setting. We propose that this is indicative of the strength of negative psychological experiences, particularly among those with SPA. Further research is required to elucidate specific differences in psychological responses across exercise settings.

A notable finding was the preference to exercise with an overweight companion in a real setting for high SPA individuals than low SPA individuals, but no preference differences in a VR exercise setting. These findings are consistent with Dunlop and Schmader’s (2014) study, where it was found that overweight individuals with high SPA reported a stronger preference to exercise with overweight companions in an exercise class compared to those with low SPA. The present investigation extends these findings across exercise settings, providing insight into preferences in VR and real-life environments. Previous research has suggested in-shape physique companions can reduce motivation and enjoyment of exercise among individuals with high SPA. However, the current results suggest that the VR-based exercise task may have minimised the anticipated psychological distress associated with real social exercise settings for those with high SPA (Crawford and Eklund 1994). The preferences across the two exercise settings may, thus, reflect differences in saliency of threat of physique evaluation (Kruisselbrink et al. 2004; Lindwall and Lindgren 2005). The finding that the physique of the exercise companion was not a deterrent in a VR exercise setting for individuals with high SPA reveals that VR may be perceived to be a less threatening or evaluative social context in which to exercise.

This study has several limitations that should be noted. Firstly, the small sample size in the high SPA group (*n* = 12) may have reduced statistical power (Haas 2012; Sheps 1993). The small group of high SPA participants was likely a result of convenience sampling, not unexpected in experimental exercise research cohorts who are generally highly active and non-anxious (Hausenblas et al. 2004). A second potential limitation is that due to current VR software limitations, the saliency of the physique of the exercise companion was weakened as it was not depicted in the VR avatars. Several methods were employed to control for this potential shortcoming in the VR system, such as the social interaction (Skype call) and presentation of a full-body picture of the exercise companion throughout the running task. Although this is a limitation in the current technology, it is representative of the capabilities of commercial VR programmes. Finally, the current study aimed to investigate the current hypotheses within a female population, and therefore, the findings are only generalisable within this population.

Drawing from the limitations, future research could specifically target individuals with high SPA, or individuals who engage in low levels of exercise for fear of evaluation. Future research could also involve collaboration with technology specialists to create a VR exercise program which allows for more visible alteration of the physique of an exercise companion, thus facilitating an improved manipulation of avatar features. Although we did ask participants about their companion preferences in different exercise settings, there were some limitations to the methods. Our preference measure was created for the purpose of the current study and had not been previously validated. In addition, it would be beneficial to experimentally compare real-world and VR exercise in individuals with high SPA and to allow participants to choose their preferred exercise setting. Given that SPA can affect a range of populations, future investigations may involve replication of this study with larger and more diverse samples. Considering the prevalence of SPA and self-presentational concerns in physical activity contexts amongst middle-aged adults (McAuley et al. 1995), young men (Martin et al. 2006), patients with specific diseases (Loney et al. 2008), and middle or high-school students (Sabiston et al. 2007), future research could be extended to these populations to determine whether the current findings generalise beyond females.

Intervention strategies to reduce the experience of SPA in an effort to increase physical activity have been efficacious for adolescent females (Lindwall and Lindgren 2005) and male and female university students (Scott 2005). Moreover, exercise video games (exergames) have been reported to significantly reduce SPA in male and female university students (Song et al. 2014). Given that exercise can reduce SPA, but that real-world exercise settings can heighten concerns and pressure for individuals with high SPA, VR-based exercise involving avatars may provide an alternative approach. Similar benefits have indeed been observed in VR-based interventions for specific-phobias, where participant preferences to engage in an intervention were greater when offered a VR exposure than an in-vivo task (Botella Arbona et al. 2014). Future research could involve a test of VR-based exercise interventions for individuals with high SPA as this may provide a less threatening exercise context than a real-world exercise setting.

In conclusion, the present study is the first to investigate the influence of the physique of an exercise companion on psychological states within a VR environment utilising an experimental design. The findings have indicated that the strength of negative psychological associations has consequences for individuals with both high and low SPA. Within an SCT framework, these negative associations are likely the result of upward comparisons which exert influence by way of the negative contrast effect. The lack of preference for physique of exercise companion in a VR setting in our sample of female exercisers is an important discovery. Considering the preference to use an in-shape appearance of avatars in popular VR programmes, our findings demonstrate that an in-shape exercise companion in a VR setting may not be an exercise deterrent for females with high SPA. Framed in this manner, VR-based exercise may be helpful among females who worry about their appearance or feel self-conscious while exercising.

## Declarations

### Data availability statement

The datasets generated during and/or analysed during the current study are available from the corresponding author on reasonable request for a period of 5 years following publication.
